# Specialized Functional Diversity and Interactions of the Na,K-ATPase

**DOI:** 10.3389/fphys.2016.00179

**Published:** 2016-05-25

**Authors:** Vladimir V. Matchkov, Igor I. Krivoi

**Affiliations:** ^1^Department of Biomedicine, Aarhus UniversityAarhus, Denmark; ^2^Department of General Physiology, St. Petersburg State UniversitySt. Petersburg, Russia

**Keywords:** Na, K-ATPase molecular heterogeneity, subcellular microdomains, cardiotonic steroids, signaling pathways, blood pressure, cell survival

## Abstract

Na,K-ATPase is a protein ubiquitously expressed in the plasma membrane of all animal cells and vitally essential for their functions. A specialized functional diversity of the Na,K-ATPase isozymes is provided by molecular heterogeneity, distinct subcellular localizations, and functional interactions with molecular environment. Studies over the last decades clearly demonstrated complex and isoform-specific reciprocal functional interactions between the Na,K-ATPase and neighboring proteins and lipids. These interactions are enabled by a spatially restricted ion homeostasis, direct protein-protein/lipid interactions, and protein kinase signaling pathways. In addition to its “classical” function in ion translocation, the Na,K-ATPase is now considered as one of the most important signaling molecules in neuronal, epithelial, skeletal, cardiac and vascular tissues. Accordingly, the Na,K-ATPase forms specialized sub-cellular multimolecular microdomains which act as receptors to circulating endogenous cardiotonic steroids (CTS) triggering a number of signaling pathways. Changes in these endogenous cardiotonic steroid levels and initiated signaling responses have significant adaptive values for tissues and whole organisms under numerous physiological and pathophysiological conditions. This review discusses recent progress in the studies of functional interactions between the Na,K-ATPase and molecular microenvironment, the Na,K-ATPase-dependent signaling pathways and their significance for diversity of cell function.

## Introduction

The Na,K-ATPase is “an enzyme of life” because of its essential role in cell life and death. The Na,K-ATPase is an ubiquitous membrane transport protein responsible for establishing and maintaining high K^+^ and low Na^+^ concentrations in the cytoplasm (Skou, 1957). This ion translocation activity underlies the resting membrane potential, excitability, and provides the driving force for secondary ion transport. Ionic homeostasis maintained by the Na,K-ATPase is critical for numerous cellular functions and processes, including cell growth, differentiation, migration, contraction, secretion, and volume regulation. The list of these cellular tasks is constantly growing. Na,K-ATPase varies in its molecular organization, exhibiting distinct properties, and localization that suggests a specialized functional diversity (Sweadner, 1989; Blanco and Mercer, 1998; Mobasheri et al., 2000; Geering, 2008; Li and Langhans, 2015). Our current knowledge suggests highly complex and isoform-specific reciprocal functional interactions and signaling between the Na,K-ATPase and neighboring proteins and lipids. Na,K-ATPase is able to form multimolecular complexes where it participates as scaffolding protein in formation of specialized sub-cellular microdomains or microcompartments (Xie and Askari, 2002; Schoner and Scheiner-Bobis, 2007; Rajasekaran et al., 2008; Reinhard et al., 2013;Krivoi, 2014).

The extracellular loops of Na,K-ATPase catalytic α subunit form unique highly specific binding site for cardiotonic steroids (CTS) and their circulating endogenous analogs (Bagrov et al., 2009; Ogawa et al., 2009; Lingrel, 2010; Laursen et al., 2013). Physiological significance of CTS binding site is still under debate. It remains uncertain whether it affects numerous cellular functions by inhibiting enzymatic activity that leads to modulation of ion homeostasis or by conformational changes of the α subunit and initiation of a signal transduction. The role of the Na,K-ATPase CTS binding site suggests its involvement in regulation of diverse cellular functions, including synaptic and neural processes (Lichtstein and Rosen, 2001; Goldstein et al., 2006, 2011; Song et al., 2013), cell survival and neuroprotection (Golden and Martin, 2006; Dvela et al., 2012; Sibarov et al., 2012; Dvela-Levitt et al., 2014), muscle contraction (Dostanic-Larson et al., 2005; Radzyukevich et al., 2009), intercellular communications (Matchkov et al., 2007, 2012), and gene expression (Xiao et al., 2002; Kulikov et al., 2007; Orlov and Hamet, 2015; Blanco and Venugopal, 2016). Knowledge on the role of Na,K-ATPase and endogenous CTS in intracellular signaling opens new perspectives for modulation of cell function under normal and pathological conditions. The present review focuses on the isoform-specific functions of the Na,K-ATPase and specialized interactions with molecular environment which underlie a variety of the Na,K-ATPase-dependent regulatory mechanisms.

## Molecular diversity of Na,K-ATPase

A molecular heterogeneity of the same functional protein is one of the well-documented principles in cell biology (Le Novère et al., 2002; Massoulié, 2002; Drabkina and Krivoi, 2004; Markov et al., 2015). It includes the heterogeneity in structure and diversity in function of the Na,K-ATPase (Sweadner, 1989; Blanco and Mercer, 1998; Mobasheri et al., 2000; Geering, 2008; Li and Langhans, 2015).

Na,K-ATPase is a P-type ATPase, a protein vital for cellular function and ubiquitously expressed in the plasma membrane of all animal cells. An enzymatic activity of the Na,K-ATPase provides an electrical excitability and driving force for many other transmembrane transports. An ion translocation, described by Post-Albers reaction, suggests cyclic transitions of the Na,K-ATPase between two principal conformational states, E1 and E2 which selectively bind three Na^+^ ions or two K^+^ ions, respectively. Each cycle uses the energy from hydrolysis of one ATP molecule. This active ion transport generates an additional negative (electrogenic) membrane potential due to the net outward transfer of one positive charge per transport cycle (Sperelakis, 2001; Dobretsov and Stimers, 2005).

Minimal functional unit of the Na,K-ATPase is a heteromeric complex consisting of a large α catalytic (~110 kDa) and smaller β glycoprotein (~31.5 kDa) subunits. The α subunit is responsible for ion transport. This subunit has 10 transmembrane domains which contain binding sites for Na^+^ ions on the extracellular loops and for K^+^ ions and ATP on the intracellular loops (Blanco and Mercer, 1998; Mobasheri et al., 2000). The β subunit is a single-transmembrane protein which is required for enzymatic activity and modulates the enzyme affinity to Na^+^ and K^+^ ions. It also functions as chaperone targeting the α subunit to the plasma membrane and plays an important role in cell adhesion (Liu and Askari, 2006; Liu et al., 2011; Tokhtaeva et al., 2012). In some tissues a small single-transmembrane protein of FXYD family (~7 kDa) has been found to be associated with the functional Na,K-ATPase α/β complexes and known to modulate enzymatic activity (Sweadner and Rael, 2000; Garty and Karlish, 2006; Geering, 2008; Pavlovic et al., 2013; Arystarkhova, 2016). Four isoforms of the α subunit and three isoforms of the β subunit are expressed in a cell- and tissue-specific manner providing wide molecular diversity of the Na,K-ATPase (Blanco and Mercer, 1998; Mobasheri et al., 2000; Mijatovic et al., 2007; Li and Langhans, 2015). Seven proteins of the FXYD family provide additional diversity to these assemblies (Sweadner and Rael, 2000; Garty and Karlish, 2006; Geering, 2008; Pavlovic et al., 2013; Arystarkhova, 2016).

It is generally accepted that the ubiquitous α1 isoform plays main “housekeeping” role while the other isoforms are expressed in a cell-specific manner. In some tissues, e.g., erythrocytes, kidney epithelia and liver, the α1 isoform is the only isoform expressed, while the majority of other cell types co-expressed other α isoforms serving additional regulatory functions that often are poorly understood. Thus, the α2 isoform is principally expressed in skeletal, cardiac and smooth muscles as well as in glial cells while the α3 isoform is characteristic for neuronal tissues (Blanco and Mercer, 1998; Mobasheri et al., 2000; Dobretsov and Stimers, 2005; Krivoi, 2012; Li and Langhans, 2015). The α4 isoform has been found only in testis (Woo et al., 2000).

## Cardiotonic steroid binding site of the Na,K-ATPase and endogenous inhibitors

All known Na,K-ATPase isozymes contain specific receptor for inhibitors collectively known as CTS—compounds characterized by a steroid nucleus. Several plants are shown to contain CTS such as ouabain, digoxin, digitoxin, and proscillaridin A (Mijatovic et al., 2007; Bagrov et al., 2009). CTS are also found in animal species and occur mainly in toads, e.g., marinobufagenin isolated from the skin of *Bufo marinus* (Bagrov et al., 2009). CTS are lethal in high concentrations while in low concentrations they (particularly, digoxin, and digitoxin) are widely used as positive inotropic agents (Gheorghiade et al., 2004).

The specific binding site for CTS is formed by an extracellular region between M1–M2, M5–M6, and M7–M8 transmembrane domains of the Na,K-ATPase α subunit (Mijatovic et al., 2007; Bagrov et al., 2009; Ogawa et al., 2009; Lingrel, 2010; Sandtner et al., 2011; Laursen et al., 2013). CTS molecules bind and stabilize the Na,K-ATPase in E2 conformation inhibiting the transport activity of the enzyme. Isoforms of the α subunit Na,K-ATPase differ in their sensitivity to ouabain, a CTS found in plants and animal tissues, with greatest difference in rodents. In rodents, the α1 isozyme is relatively resistant to ouabain (the IC_50_ values for inhibition of the Na,K-ATPase are between tens to hundreds micromolar), while the α2, α3, and α4 isozymes are two-four orders of magnitude more sensitive (Dobretsov and Stimers, 2005; Lingrel, 2010). The sensitivity to ouabain is determined by two amino acids at the positions 111 and 122 in transmembrane domains M1–M2. Genetic manipulations substituting these amino acids in mice produced mice with various combinations of the α1 and α2 isozyme sensitivities to ouabain and to study the physiological role of the CTS binding site (Lingrel, 2010).

Knowledge on the Na,K-ATPase sensitivity to ouabain and other CTS is of a great importance. Notably, apart from rodents, the α1 Na,K-ATPase isozyme in rabbit, pig, dog, sheep, guinea pig, and human is relatively sensitive to ouabain (see for review: Blanco and Mercer, 1998; Dobretsov and Stimers, 2005; Mijatovic et al., 2007; Lingrel, 2010). Some studies in humans showed high and similar affinity of α1, α2, and α3 isozymes for cardiac glycosides with ouabain binding constants in nanomolar concentration range (Wang et al., 2001). Moderate selectivity between human α-subunit isoforms was also shown. By contrast, digoxin and a number of other CTS demonstrated lower affinities and more significant selectivity compared to ouabain (Crambert et al., 2000; Katz et al., 2010; Cherniavsky Lev et al., 2015). The reasons for this differentiated selectivity remain to be elucidated.

The presence of an endogenous ouabain-like compounds was suggested almost 40 years ago (Haddy and Overbeck, 1976; Blaustein, 1977) and endogenous ouabain was later purified from human blood plasma (Hamlyn et al., 1991). Several facts point toward ouabain being a hormone synthesized and secreted by the adrenal cortex. First, ouabain has been found in high concentrations in the adrenal cortex (Hamlyn et al., 1991; Blaustein, 1993; Hamlyn, 1998; Li et al., 1998). Second, bovine adrenocortical cells have been shown to secrete ouabain in amounts greater than their storage capacity under *in vitro* conditions (Laredo et al., 1994, 1995). Third, the concentration of ouabain in adrenal venous blood is significantly higher than in arterial plasma (Boulanger et al., 1993). Moreover, adrenal cortex tumors have been characterized by overproduction and secretion of ouabain (Komiyama et al., 1999). Consistently, administration of anti-ouabain antibodies to rats produces adrenal cortex enlargement, further implicating the adrenal gland as a source of ouabain (Nesher et al., 2009).

Ouabain is believed to be synthesized in zona glomerulosa cells of the adrenal cortex, as other adrenal steroids and the synthesis involves a rate-limiting side chain cleavage of cholesterol (Laredo et al., 1995). Hydroxycholesterol, pregnenalone, and progesterone have been shown to increase the secretion of ouabain, possibly acting as precursors in its biosynthetic pathway (Hamlyn et al., 1998; Lichtstein et al., 1998). Moreover, the synthesis of ouabain follows a similar pathway as aldosterone (Hamlyn et al., 2003). However, the exact mechanisms and precursors involved in ouabain biosynthesis are still unclear. Also hypothalamus has been suggested to synthesize ouabain or ouabain-like compound(s) (Li et al., 1998) which may play a central neuromodulatory role leading to excitation of the central sympathoexcitatory pathways (Blaustein et al., 2012).

In addition to ouabain, structurally similar digoxin, marinobufogenin and a number of other digitalis-like compounds were further identified endogenously (Lichtstein and Rosen, 2001; Schoner and Scheiner-Bobis, 2007; Bagrov et al., 2009). Endogenous ouabain and marinobufagenin are well-studied. Their concentration in blood plasma and cerebro-spinal fluid of mammals varies at subnanomolar range. Elevated level of endogenous CTS has been found under physiological (e.g., strenuous exercise, newborn infants) and pathophysiological (e.g., congestive heart failure, hypertension, chronic renal failure, preeclampsia, and affective disorders) conditions suggesting the role for these compounds for modulation of body function and in pathologies (Lichtstein and Rosen, 2001; Schoner, 2002; Dobretsov and Stimers, 2005; Bagrov et al., 2009).

## Subcellular compartmentalization of the Na,K-ATPase function

Subcellular compartmentalization is one of the basic principles of intracellular organization (Saks et al., 2009). Particularly diffusion restriction between cytosolic bulk and spatially limited submembrane space is enabled in local microdomains. In cardiac cell, the α1 Na,K-ATPase isozyme is relatively uniformly distributed between external sarcolemma and T-tubular membranes, while the α2 isozyme is concentrated in T-tubules with a preferential localization close to the junctional sarcoplasmic reticulum (SR) (Shattock et al., 2015). In addition, the Na,K-ATPase forms large membrane macromolecular complexes with Na^+^,Ca^2+^ exchanger (NCX), and ATP-sensitive K^+^ (K_ATP_) channels, coordinated by ankyrin-B (Li et al., 2010). The K_ATP_ channels are densely expressed at the places where T-tubules create membrane junctions with the SR (Alekseev et al., 2012). Being co-localized with the Na,K-ATPase, K_ATP_ channels open in response to increased energy utilization accompanied by the local ATP depletion within a diffusion-restricted submembrane space. This adjusts cardiac cell electrogenesis and excitability across a wide range of workloads (Kabakov, 1998; Alekseev et al., 2012). Similar close functional interaction between the α2 Na,K-ATPase isozyme and the K_ATP_ channels was shown in vascular smooth muscle cells (Glavind-Kristensen et al., 2004; Matchkov et al., 2007).

In skeletal muscle cells, the α1 and α2 Na,K-ATPase isozymes also have distinct distributions and membrane localization. The α1 isozyme comprises up to 40% of total Na,K-ATPase and is expressed only on the outer plasma membrane. The α2 isozyme comprises 60–80% of total Na,K-ATPase content (Orlowski and Lingrel, 1988; He et al., 2001) and the majority of the α2 isozyme is expressed in the interior transverse tubule membranes, with smaller pools localized to the end-plate membrane and surface caveolae (Williams et al., 2001; Cougnon et al., 2002; Heiny et al., 2010; Kristensen and Juel, 2010). Diffusion alone is not sufficient to remove the excitation-related K^+^ load in the T-tubules, the concentration of which, reaches tens of millimolar (Sejersted and Sjogaard, 2000; Clausen, 2013). The existence of two α isozymes of the Na,K-ATPase with distinct locations and K^+^ affinities was proposed (DiFranco et al., 2015). The α1 isozyme with relatively high K^+^ affinity mediates most of the basal Na^+^ and K^+^ ion transport and plays a major role in setting resting transmembrane ion gradients and resting membrane potential. Located in T-tubules α2 isozyme with apparently low K^+^ affinity operates substantially below its maximum capacity in the resting muscles, but its activity can rapidly increase during membrane excitation and K^+^ accumulation. This helps to maintain muscle excitability, contraction, and oppose fatigue.

The Na,K-ATPase containing membrane microdomains were shown to be associated to the “junctional” SR and include the NCX as one of the key functional players (Moore et al., 1993; Juhaszova and Blaustein, 1997; Golovina et al., 2003; Lynch et al., 2008). These microdomains were shown in a variety of cell types including neurons, glia, and myocytes. Other membrane proteins were also shown to co-localize in these microdomains, e.g., diverse plasma membrane Ca^2+^ channels, sarco/endoplasmic reticulum Ca^2+^-ATPase (SERCA), sarcoplasmic reticulum IP_3_, and ryanodine receptors (Blaustein and Golovina, 2001; Blaustein, 2013). In these specialized microdomains, called “PLasmERosome,” the Na,K-ATPase and its molecular environment function cooperatively to regulate locally the intracellular Ca^2+^ signaling (Blaustein and Golovina, 2001; Blaustein, 2013). The plasma membrane and SR components appear to be linked through the cytoskeletal spectrin network and adaptor protein, ankyrin 2 (Lencesova et al., 2004). These microdomains specifically contained ouabain-sensitive α2 or α3 Na,K-ATPase isozymes. The N-terminal sequence targets and tethers the α2 Na,K-ATPase isozyme to its specific localization in the plasma membrane (Song et al., 2006).

Restriction of Na^+^ and Ca^2+^ diffusion enables the appearance of concentration gradients between these restricted spaces and the bulk cytosol. Interestingly, the α2 Na,K-ATPase isozyme have much lower affinities for Na^+^ than the α1 isozyme (Zahler et al., 1997). This suggests that intracellular Na^+^ will rise more in the restricted spaces controlled by the α2 Na,K-ATPase isozyme than the global intracellular Na^+^ which is under α1 isozyme control. Thus, these α2-isozyme-associated-microdomains are well-organized to control the local Na^+^ electrochemical gradient which can influence Ca^2+^ homeostasis via the co-localized NCX isoform 1 (Golovina et al., 2003; Lynch et al., 2008). This links cellular Ca^2+^ concentration to Na^+^ concentration; a spatially restricted rise in Na^+^ will lead to a localized elevation of intracellular Ca^2+^. Such interactions in ion metabolism do not only control local Ca^2+^ but also affect global intracellular Ca^2+^ via modulation of the SR load. This interaction generally explains the well-known potentiating effect of ouabain on vascular contraction (Miriel et al., 1999; Iwamoto et al., 2004; Zhang et al., 2005, 2010; Blaustein and Wier, 2007).

Alterations in the Na,K-ATPase activity will change the cellular Ca^2+^ homeostasis and enhance loading of intracellular Ca^2+^ stores (Golovina et al., 2003). This has normally been considered a result of the elevation in the intracellular Na^+^ concentration slowing the clearance of Ca^2+^ by NCX and, therefore, allowing extra Ca^2+^ pumped into the stores (see above). The reduction of α2 isozyme activity (either by knocking it down or by pharmacological inhibition) should therefore, be associated with elevated contractility of vascular smooth muscle cells, although this is not always the case. It has been previously reported that, in contrast to the effect of ouabain, a transient siRNA-induced downregulation of the α2 Na,K-ATPase isozyme suppresses contractile responses of rat mesenteric small arteries (Matchkov et al., 2012). Although ouabain increased sensitivity to the contractile stimuli in control arteries, it had no effect on the α2-isozyme-downregulated arteries. Surprisingly, the reduced expression of α2 isozyme was associated with higher intracellular Ca^2+^ concentration but suppressed contractile response of the arterial wall (Matchkov et al., 2012). This suggests that downregulation of the α2 Na,K-ATPase isozyme led to reduction in Ca^2+^-sensitivity of vascular smooth muscle cell contractile machinery.

These surprising findings might seem to be contrasting to other reports where small arteries from α2-isozyme-knockout mice showed an increased myogenic tone (Iwamoto et al., 2004; Shelly et al., 2004; Dostanic et al., 2005; Zhang et al., 2005). However, the α2-isozyme-downregulated arteries also had increased myogenic contraction possibly due to elevated basal intracellular Ca^2+^ which might be a consequence of coordinated reduction in the NCX expression (Matchkov et al., 2012). Stimulation with agonists was, however, less effective in the α2-isozyme-downregulated arteries where both sensitization to Ca^2+^ and Ca^2+^ release through the IP_3_ receptors were suppressed. The reason for this compromised agonist-induced Ca^2+^ sensitivity in the arteries with reduced α2 Na,K-ATPase isozyme expression is not known but it suggests a more complex mechanism for the control of smooth muscle contractility by the Na,K-ATPase than the modulation of intracellular Ca^2+^ concentration via membrane potential (Mulvany et al., 1984; Aalkjaer and Mulvany, 1985) and ion homeostasis (Golovina et al., 2003; Lynch et al., 2008).

A direct interaction of the α Na,K-ATPase subunit N-terminus with IP_3_ receptor has been established suggesting that ouabain-induced conformational changes in the α subunit can directly liberate Ca^2+^ from intracellular depot (Aizman et al., 2001; Zhang et al., 2006; Tian and Xie, 2008). Importantly, ouabain-evoked Ca^2+^ signaling can not only affect the contractility of cardiac and smooth muscles but also regulates via the Ca^2+^-sensitive transcription factors protein expression, cell proliferation, and differentiation (Aizman et al., 2001; Fontana et al., 2013).

In membrane microdomains the Na,K-ATPase is shown to be organized together with interacting proteins in signalosome (Aydemir-Koksoy et al., 2001; Wang et al., 2004; Efendiev et al., 2005; Tian et al., 2006) restricted to caveolae (Wang et al., 2004; Liu and Askari, 2006; Schoner and Scheiner-Bobis, 2007; Tian and Xie, 2008; Liu et al., 2011; Morrill et al., 2012). Caveolin is a protein essential for caveolae formation and direct Na,K-ATPase/caveolin interaction was previously shown (Wang et al., 2004; Cai et al., 2008; Heiny et al., 2010; Morrill et al., 2012). Cholesterol-rich membrane microdomains, i.e., lipid rafts and caveolae, are nanoscale assemblies of sphingolipid, cholesterol, and proteins that form platforms for subcellular signaling and trafficking (Razani et al., 2002; Lingwood and Simons, 2010; Harvey and Calaghan, 2012; Sebastiao et al., 2013). The formation of cholesterol rich lipid microdomains is important for Na,K-ATPase targeting and regulation and the reciprocal interactions between the Na,K-ATPase and cholesterol were proposed (Cornelius, 2008; Chen et al., 2011; Kapri-Pardes et al., 2011; Haviv et al., 2013; Cornelius et al., 2015).

## The Na,K-ATPase/Src signaling pathway

In the functional signalosome the Na,K-ATPase has been suggested to interact and regulate protein kinases as well as function as scaffold protein for receptors and effectors (Li and Xie, 2009). The experimental findings during the last decade suggest that the Na,K-ATPase can function as an important signal transducer (Aizman and Aperia, 2003; Li et al., 2009; Liu and Xie, 2010). Indeed, two functionally separate pools of the Na,K-ATPase have been suggested to be engaged in the “classic” ion transport and cellular activities other than ion pumping (Xie et al., 2015).

The unconventional non-pumping Na,K-ATPase resides in restricted membrane microdomains, where it directly interacts with protein kinases, ion channels, and transporters (Xie, 2003; Xie and Cai, 2003). Thus, it has been shown that the central loop of Na,K-ATPase interacts with phospholipase C-γ (PLCγ) and the N-terminus binds to IP_3_ receptors (Yuan et al., 2005). This signalosome comprises also several anchoring proteins, Src kinase and has been shown to be an important modulator of intracellular Ca^2+^ signal (Haas et al., 2000; Liu et al., 2000). Ouabain can act through this signalosome in two synergistic manners (Yuan et al., 2005). First, it can force PLCγ and IP_3_ receptors into close proximity and facilitate the signal transmission. Second, an activation of the Na,K-ATPase-associated Src could lead to tyrosine phosphorylation of both PLCγ and the IP_3_ receptors that will sensitize the receptor to IP_3_ produced by PLCγ. In this term, the Na,K-ATPase-Src interaction is important not only for ouabain signaling but also for many other agonist-induced intracellular responses that involve IP_3_ signaling and tyrosine phosphorylation in general. Binding of ouabain to the Na,K-ATPase releases Src kinase that can affect intracellular Ca^2+^ as well as modulate other signaling pathways including gene expression (Xie and Cai, 2003).

Although there is some controversy regarding an interaction between the Na,K-ATPase and Src kinase, an activation of Src kinase by phosphorylation is a well-established response to submicromolar concentrations of ouabain. Several studies suggest that the Na,K-ATPase-associated Src kinase specifically activated by ouabain (Haas et al., 2000; Liang et al., 2006; Tian et al., 2006; Li et al., 2009; Lai et al., 2013; Ye et al., 2013; Banerjee et al., 2015). This signaling microdomain model disagreed with other studies suggesting that ouabain-induced Src kinase activation is a result of the ATP-sparing effect of the Na,K-ATPase inhibitor on these two enzymes competing for ATP (Weigand et al., 2012; Gable et al., 2014). However, some skeletal muscle studies showed that submicromolar and micromolar concentrations of ouabain do not affect the global intracellular ATP/ADP ratio while significant phosphorylation of Src kinase and its activation were observed (Kotova et al., 2006a,b). Nevertheless, these studies addressed the global ATP/ADP ratio, and there is a possibility for spatially restricted changes in the concentrations. Accordingly, it has been shown that the Na,K-ATPase-dependent Src kinase activity is maintained in cells expressing a non-pumping mutant of rat α1 isoform (Liang et al., 2006). The involvement of Na,K-ATPase in signaling cascade does not exclude a role for its ion pumping function in ouabain-induced effects. Moreover, since intracellular Na^+^ ions regulate the conformation of the Na,K-ATPase (e.g., the E1 state), it is possible that changes in intracellular Na^+^ concentration could also regulate the formation of the Na,K-ATPase/Src complex, and thus cellular Src activity (Li et al., 2009). This Na,K-ATPase-dependent Src kinase signaling is hypothesized to modulate arterial contraction and blood pressure as discussed below.

## Na,K-ATPase and hypertension

The correlation between circulating ouabain and blood pressure was suggested almost 35 years ago (Hamlyn et al., 1982). Almost 50% of patients with uncomplicated essential hypertension have been reported to have elevated endogenous ouabain (Rossi et al., 1995). In accordance with hemodynamic background of hypertension characterized by an increase in peripheral resistance and unchanged cardiac output, plasma ouabain level correlates positively with elevated peripheral resistance and left ventricular hypertrophy, but not with cardiac output (Manunta et al., 1999; Pierdomenico et al., 2001). Also other endogenous CTS correlate with blood pressure, e.g., urinary marinobufagenin level increases with elevated blood pressure and aortic stiffness in patients (Jablonski et al., 2013).

These human studies received further strong experimental support from several animal models of ouabain-dependent hypertension. Chronic administration of ouabain, leading to an increase of its plasma concentration to the level observed in essential hypertension, produced hypertension in rats (Yuan et al., 1993; Manunta et al., 2000; Pulgar et al., 2013). This ouabain-induced hypertension was associated with elevated peripheral vascular resistance that is a result of inward arterial structural remodeling (Briones et al., 2006) and increased contractility of the resistance arteries (Pulgar et al., 2013). Endogenous ouabain-like inhibitor of the Na,K-ATPase was also implicated in pulmonary hypertension (Janssens et al., 1993). Plasma ouabain level is elevated in several other rodent models of hypertension, including DOCA-salt, reduced renal mass, Milan hypertensive rats, Dahl S rats on high-salt diet, and adrenocorticotropic hormone induced hypertension (for review see: Blaustein et al., 2012).

Inhibition of endogenous ouabain action by systemic administration of ouabain antagonist rostafuroxin or digibind (an antibody to endogenous ouabain) lowers blood pressure and even prevents hypertension in the high-ouabain hypertension models (Dostanic-Larson et al., 2005; Manunta et al., 2006). Finally, knock-in of ouabain-resistant mutation of the α2 Na,K-ATPase isozyme prevents ouabain-induced hypertension (Dostanic et al., 2005; Dostanic-Larson et al., 2005; Lorenz et al., 2008). This indicates the importance of the α2 isozyme in pathogenesis of hypertension. The importance of the α1 Na,K-ATPase isozyme was, however, also suggested. Thus, blood pressure was shown to correlate with the expression of either α1 or α2 isozyme (Pritchard et al., 2007). However, these two isozymes have coordinated expression profiles, where overexpression of one isozyme increased the expression of another. This makes it therefore difficult to distinguish their specific roles, although it had been concluded that the α2 Na,K-ATPase isozyme seems to play more significant role (Pritchard et al., 2007).

Presently, there is no generally accepted molecular mechanism which could explain how the inhibition of Na,K-ATPase leads to an elevation of blood pressure. The situation is further complicated by the fact that not all CTS have a similar effect on blood pressure. Thus, in contrast to ouabain, digoxin does not raise blood pressure and has even antihypertensive action in ouabain-dependent hypertension models (Manunta et al., 2000; Zulian et al., 2013). Nevertheless, all known “classic” CTS inhibit the Na,K-ATPase pumping activity and exert vasotonic effects *in vitro*; however, digoxin-like steroids can antagonize the vasotonic effects of ouabain-like steroids, and vice versa (Song et al., 2014). This phenomenon of ouabain-digoxin antagonism is not unique for blood vessels and is also characteristic for glutamate-induced Ca^2+^-transients in primary cultured hippocampal neurons (Song et al., 2014).

Ouabain-digoxin antagonism might be a result of functional selectivity or biased signaling of the Na,K-ATPase as it is known for some G-protein-coupled receptors (Kenakin, 2011; Kenakin and Christopoulos, 2013). Thus, the possibility for different conformational changes of the Na,K-ATPase upon a binding of different CTS has been hypothesized to be the reason for different functional effects in spite of their uniform inhibitory action. However, recent crystal structure analyses of the high-affinity Na,K-ATPase-ouabain and -digoxin complexes do not support this possibility, although they do not exclude it (Laursen et al., 2013, 2015).

It has been suggested that the Na,K-ATPase functions as a tetraprotomer (Hah et al., 1985) where single CTS blocks all pumping activity but digoxin-like steroids are able to reactivate the ouabain-inhibited tetraprotomers via de-oligomerization (Song et al., 2014). This antagonism is shown for resistance arteries *in vitro*. However, ouabain-like steroids elevate blood pressure while digoxin-like steroids do not and even antagonize the effect of ouabain (Manunta et al., 2000; Zulian et al., 2013). Thus, only one direction of ouabain-digoxin antagonism can be seen *in vivo* in contrast to *in vitro* experiments (Song et al., 2014). Low constitutive level of endogenous ouabain was implicated in this inconsistency.

## Modulation of arterial contractility by the Na,K-ATPase

*In vitro* studies suggest that many of the functional and structural alterations in arteries from hypertensive animals could be consequences of elevated plasma ouabain (Blaustein et al., 2012). The Na,K-ATPase has a significant role in regulation of vascular tone and contractility, and therefore has been proposed to modulate peripheral vascular resistance and blood pressure (Blaustein and Wier, 2007). Two α-isozymes of the Na,K-ATPase are expressed in vascular smooth muscles, where the α1 Na,K-ATPase isozyme is homogeneously distributed over the cell membrane while the α2 isozyme has a spatially restricted distribution (Lee et al., 2006; Matchkov, 2010).

Activation of the Na,K-ATPase results in membrane hyperpolarization and vascular relaxation while its inhibition causes membrane depolarization and vascular contraction. Inhibition of the Na,K-ATPase by ouabain in a broad concentration range between 0.01 μM and 1 mM had no effect on resting vascular tone while it significantly potentiated the agonist-induced contraction (Aalkjaer and Mulvany, 1985). Accordingly, ouabain produced acute and transient (within 10 min) concentration-dependent depolarization of smooth muscles in both resting and agonist-stimulated arteries (Nilsson and Mulvany, 1981; Mulvany et al., 1982; Aalkjaer and Mulvany, 1985). At the same time, a prolonged exposure to micromolar concentrations of ouabain suppressed arterial contractility (Nilsson et al., 2001). The reason for these short-term potentiating and long-term depressive effects of arterial contraction is unknown. It has been suggested that under physiological conditions ouabain-induced depolarization and the following voltage-dependent Ca^2+^ influx play an important role in the potentiation of arterial contraction, although the role of intracellular Na^+^ for the NCX modulation should not be underestimated (Mulvany et al., 1984; Aalkjaer and Mulvany, 1985). Simultaneous analyses of contraction and intracellular Ca^2+^ concentration changes demonstrated also that the long-term depressive effect of ouabain on arterial contraction was due to desensitization of smooth muscle contractile machinery to Ca^2+^ ions (Nilsson et al., 2001).

The discussed above hypothesis about Na,K-ATPase-dependent Src kinase signaling is mostly based on the studies with epithelial cells (Aizman and Aperia, 2003; Yuan et al., 2005; Li et al., 2009; Liu and Xie, 2010; Lai et al., 2013). However, previous study showed opposite effects of two Na,K-ATPase inhibitors, ouabain and digoxin, on blood pressure (Manunta et al., 2000). It has been suggested that although both of inhibitors block pumping activity of the Na,K-ATPase, only ouabain elevates intracellular Ca^2+^ through an activation of the Src kinase (Zulian et al., 2013). Digoxin, which is unable to activate Src kinase, fails to potentiate smooth muscle contraction. Thus, this strongly suggests the importance of the Na,K-ATPase-Src signaling pathway in regulation of arterial tone and suggests that hypertensive action of endogenous ouabain-like steroids is mediated via Src kinase signaling. This is in accordance with the functional study on isolated arterial segments showing the importance of Src signaling for vascular tone control (Toma et al., 1995).

Digoxin-related synthetic steroid, rostafuroxin (Quadri et al., 1997) does not affect pumping activity of the Na,K-ATPase (Ferrari et al., 1998) and has itself no effect on the arterial tone (Zhang et al., 2005). It antagonizes, however, the vasotonic action of ouabain-like CTS (Song et al., 2014) and hypertension associated with an elevation of endogenous ouabain level (Ferrari, 2010). This antihypertensive effect of rostafuroxin has been associated with suppression of ouabain-induced Src-kinase-dependent signaling pathway (Wenceslau and Rossoni, 2014).

The importance of the α1 Na,K-ATPase isozyme for initiation of the Src kinase signaling has been shown (Xie et al., 2015), although whether this is also the case for vascular smooth muscle cells remains to be identified. The specific Src-kinase-dependent pathways upon ouabain binding by the Na,K-ATPase remain to be elucidated but this signaling was shown in several studies with smooth muscle cells in culture (Haas et al., 2000, 2002; Liu et al., 2004). Activation of Src kinase triggers Src-dependent phosphorylation of epidermal growth factor receptor and an activation of Ras/MAPK (mitogen-activated protein kinase) cascade (Haas et al., 2000, 2002) as well as numerous other signaling pathways important for vascular smooth muscle function and phenotype (for review: MacKay and Knock, 2015). Src kinases in smooth muscles were shown to be involved in reactive oxygen species signaling (Akhand et al., 1999; Giannoni et al., 2005; Knock and Ward, 2011), G-protein-coupled receptor stimulations (Luttrell and Luttrell, 2004), tyrosine phosphorylation of transient receptor potential channels (Kawasaki et al., 2006), voltage-gated Ca^2+^ channels (Wijetunge et al., 2000; Gui et al., 2010) and K^+^ channels (Alioua et al., 2002; Sung et al., 2013), modulation of Rho pathways (Guilluy et al., 2010; Gadepalli et al., 2012) and myosin phosphatase activity (Velasco et al., 2002). These signaling pathways will affect intracellular Ca^2+^ concentration and sensitization of contractile machinery to Ca^2+^, modulate proliferation and apoptosis suggesting their role in vascular repair and remodeling.

## Other Na,K-ATPase dependent signaling pathways in the vascular wall

In addition to the Src kinase signaling, the importance of which still needs to be validated for the vasculature, the Na,K-ATPase has been shown to interact in the arterial wall with several other signaling pathways. Thus, the Na,K-ATPase associates with salt-inducible kinase 1 (SIK1), a sucrose-non-fermenting-like isoform of the 5′-adenosine monophosphate-activated protein kinase (AMPK) family (Sjostrom et al., 2007). A Ca^2+^/calmodulin-dependent activation of SIK1 results in the de-phosphorylation of the α subunit Na,K-ATPase and an increase its catalytic activity. This pathway is shown to be activated by high salt intake in both kidneys (Sjostrom et al., 2007) and human vascular smooth muscle cells (Popov et al., 2011). Interestingly, a single nucleotide polymorphism of SIK1 has associated with low blood pressure and decreased left ventricle mass suggesting the importance of this signaling for blood pressure control (Popov et al., 2011).

Glutathionylation of β1 subunit of the Na,K-ATPase is an important pathway to modulate Na,K-ATPase activity by physiological and pathophysiological stimuli. Thus, angiotensin II has previously been shown to inhibit the Na,K-ATPase in vascular smooth muscle cells via NADPH oxidase-dependent glutathionylation of β1 subunit suggesting the involvement of this pathway in elevation of arterial tone and angiotensin-induced hypertension (Liu et al., 2013). Importantly, this action was antagonized by FXYD proteins showing their important vascular protective role under oxidative stress (Liu et al., 2013). The glutathionylation pathway has also been shown to play an important role in agonist-induced inhibition of the Na,K-ATPase activity in smooth muscle cells (Dey et al., 2013). An antagonistic action of FXYD1 protein, phospholemman (PLM) was associated with de-glutathionylation of the Na,K-ATPase and has been suggested to be modulated via protein kinase C (PKC) phosphorylation (Dey et al., 2012). This PKC mediated signaling stimulates the Na,K-ATPase turnover without affecting affinity for Na^+^. In pulmonary artery wall, PKC was also implemented in Na,K-ATPase inhibition by HETE-20, a cytochrome P-450 metabolite of arachidonic acid (Singh et al., 2012). This action explains a moderate potentiation of vascular tone by arachidonic acid. Finally, PKC is involved in modulation of the α2 Na,K-ATPase isozyme upon adrenoceptor stimulation while the α1 Na,K-ATPase isozyme is regulated by β-adrenoceptor-dependent protein kinase A signaling (Gao et al., 1999).

## Control of intercellular coupling by the Na,K-ATPase

Na,K-ATPase is involved in modulation of vascular tone by endothelium (Edwards et al., 1998; Dora and Garland, 2001; Wenceslau and Rossoni, 2014; Hangaard et al., 2015). Hyperpolarization of endothelial cells by chemical or mechanical excitation facilitates Ca^2+^ influx which, in this way, enhances the production of endothelium-derived relaxing factors. Besides nitric oxide (NO) and prostanoids the endothelium-dependent relaxation is mediated by an endothelium-dependent hyperpolarizing factor (EDHF) (Sandow, 2004; Edwards et al., 2010). Vasodilator effects of EDHF are strongly associated with the subjacent smooth muscle cell hyperpolarization but its nature remains controversial. It is, however, well-established that EDHF is critically dependent on endothelial Ca^2+^-activated K^+^ channels, K_Ca_2.3 and K_Ca_3.1 (Coleman et al., 2004; Sandow, 2004). Strong experimental data indicates also the significance of myoendothelial gap junctions (MEGJs) and the Na,K-ATPase in EDHF (Edwards et al., 1998; de Wit et al., 2006; Dora et al., 2008; Hangaard et al., 2015).

The heterogeneous nature of signals could be the reason for different EDHF properties depending on the type of vascular bed and experimental conditions. Thus, in rat mesenteric arteries EDHF can only in part be explained by MEGJs signaling (Edwards et al., 1998). It has been suggested that under these conditions K^+^ efflux through the Ca^2+^-activated intermittent conductance K^+^ channels (e.g., K_Ca_3.1) increases near myoendothelial projections local K^+^ concentration (K^+^ “cloud”) which, in turn, activates on the Na,K-ATPase in smooth muscle cell membrane (and inward-rectifying K^+^ channels) providing hyperpolarization of the subjacent smooth muscle cells. Which catalytic subunit of the Na,K-ATPase is important for this signal is under debate but pharmacological profile (Dora and Garland, 2001; Dora et al., 2008) and a high extracellular K^+^ saturation (McCarron and Halpern, 1990; Blanco and Mercer, 1998) suggested a major importance of the α2 Na,K-ATPase isozyme (Longden and Nelson, 2015). Moreover, it has been suggested a specific localization of the α2 Na,K-ATPase isozyme in myoendothelial projections in a close proximity with Ca^2+^-sensing receptor (CaSR) and K_Ca_3.1 (Dora et al., 2008).

It has been suggested that EDHF signals differentiate between MEGJs and K^+^ “cloud” by CaSR action on the K_Ca_3.1 channels via protein kinase A pathway (Dora et al., 2008; Hangaard et al., 2015). This differentiation is overlapping since the Na,K-ATPase activity has been shown to modulate intercellular coupling, including MEGJs (Martin et al., 2003; Matchkov et al., 2007, 2012). It has been shown that a spatially restricted microdomain of the Na,K-ATPase and the NCX (Matchkov et al., 2007) modulates intercellular communications between smooth muscle cells via controlling local intracellular Ca^2+^ homeostasis. Moreover, a physical interaction in the membrane microdomain between the α2 Na,K-ATPase isozyme, NCX and gap junction protein, connexin43 was shown (Matchkov, 2010). The role of α2 Na,K-ATPase isozyme was further validated by siRNA-induced downregulation and importance of this signaling for MEGJs was shown (Matchkov et al., 2012). In accordance with the suggested role of Na,K-ATPase for intercellular coupling pharmacological inhibition with ouabain and downregulation of the α2 Na,K-ATPase isozyme suppressed intercellular coupling and inhibited EDHF response in arteries (Matchkov et al., 2007, 2012).

Signaling pathway between the Na,K-ATPase and gap junctions is unclear. We suggested previously a Ca^2+^-dependence of uncoupling action of ouabain (Matchkov et al., 2007); however, the involvement of other signaling molecules cannot be excluded. Thus, intracellular Ca^2+^ ions can modulate gap junction directly (Enkvist and McCarthy, 1994; Schirrmacher et al., 1996; Thimm et al., 2005) and via Ca^2+^-dependent protein kinase pathways (Chuderland and Seger, 2008; Chuderland et al., 2008). Three to four types of connexins form gap junctions in the vascular wall (Gustafsson et al., 2003; Matchkov et al., 2006) but connexin43 is a suitable candidate for the regulation via Na,K-ATPase signaling. Connexin43 expressed in cultured smooth muscle cells, A7r5 (Moore et al., 1991) where this signaling is also shown and is one of the most heavily regulated gap junction proteins. It has been shown to be regulated by intracellular Ca^2+^ concentration and a broad range of intracellular signaling pathways (Lampe and Lau, 2004). Other connexin isoforms also cannot be excluded.

## Bi-modal vascular effects of ouabain

Importantly, vascular effects of ouabain can be subdivided to acute and chronic responses. In contrast to acute responses, chronic manipulations with the Na,K-ATPase have been shown to affect the expression of membrane proteins involved in Ca^2+^ transport, e.g., the NCX and C-type transmembrane receptor potential protein-6 (TRPC6) (Pulina et al., 2010; Matchkov et al., 2012; Chen et al., 2015). These expressional effects were suggested to be mediated by the α2 Na,K-ATPase isozyme via an initiation of protein kinase signaling cascade, including the Src kinase pathway. Accordingly, pharmacological inhibition of tyrosine phosphorylation prevented the expressional effects of chronic ouabain treatment (Zulian et al., 2013) but did not affect vascular responses to acute ouabain (Song et al., 2014). Downstream signalings from Src kinase activation, e.g., extracellular signal-regulated kinases 1/2 (Erk1/2) and p38, have been shown to modulate protein expression and affect cellular phenotype (Haas et al., 2000, 2002; Aizman and Aperia, 2003; Nguyen et al., 2007; Li et al., 2009; Wang et al., 2014, 2015). The involvement of other signaling pathways in the modulation of cellular phenotype cannot be excluded (Liu et al., 2007; Wu et al., 2013).

The structural remodeling of resistance arteries is an essential characteristic of hypertension (Heagerty et al., 1993; Mulvany, 1993, 2002). The functional link between arterial wall thickening and/or lumen narrowing and elevated level of endogenous ouabain-like steroids is unresolved. Ouabain has been shown to promote cell growth, proliferation and migration (Atkinson et al., 1983; Aydemir-Koksoy et al., 2001; Abramowitz et al., 2003; Allen et al., 2003; Liu et al., 2004, 2014; Schoner and Scheiner-Bobis, 2007). This action of ouabain has been suggested to be mediated through both changes in ion homeostasis and intracellular signaling pathways (Blaustein et al., 2012). Obviously, an identification of these pathways involved in structural remodeling is one of the central questions in future strategies of hypertension treatment; but, unfortunately, this was not in the scope of the majority of previous studies.

## Na,K-ATPase and skeletal muscle motor activity

The Na,K-ATPase is obligatory for excitability, electrogenesis, and contractility of skeletal muscles (Sejersted and Sjogaard, 2000; Clausen, 2008, 2013) which express the α1 and α2 Na,K-ATPase isoforms (Orlowski and Lingrel, 1988). The α2 Na,K-ATPase isozyme specifically enables working muscles to maintain contraction and resist fatigue (Radzyukevich et al., 2004, 2013; Heiny et al., 2010; DiFranco et al., 2015; Kravtsova et al., 2016). Skeletal muscle activity strongly upregulates the content of Na,K-ATPase although the α1 and α2 isozymes are regulated differently (Yuan et al., 2007; Clausen, 2008; Kristensen et al., 2008; Murphy et al., 2008; Juel, 2009; Nordsborg et al., 2009). Accordingly, physical inactivity of skeletal muscle induced by functional unloading (disuse) reduces the content of Na,K-ATPase (Clausen, 2008). However, the isoform-specificity of these changes was not studied in details.

Mechanical unloading of skeletal muscle under bed rest, joint immobilization, spinal cord injury, or other forms of muscle disuse leads to loss of muscle mass and functional decline (Baldwin et al., 2013; Bodine, 2013; Brooks and Myburgh, 2014). Weightless conditions during space flight and microgravity are also known to induce similar adaptations in skeletal muscles with the largest effect seen in postural muscles such as soleus (Fitts et al., 2013). Importantly, molecular and cellular mechanisms of disuse-induced atrophy are not completely understood (Baldwin et al., 2013; Bodine, 2013; Brooks and Myburgh, 2014).

**Figure 1:**
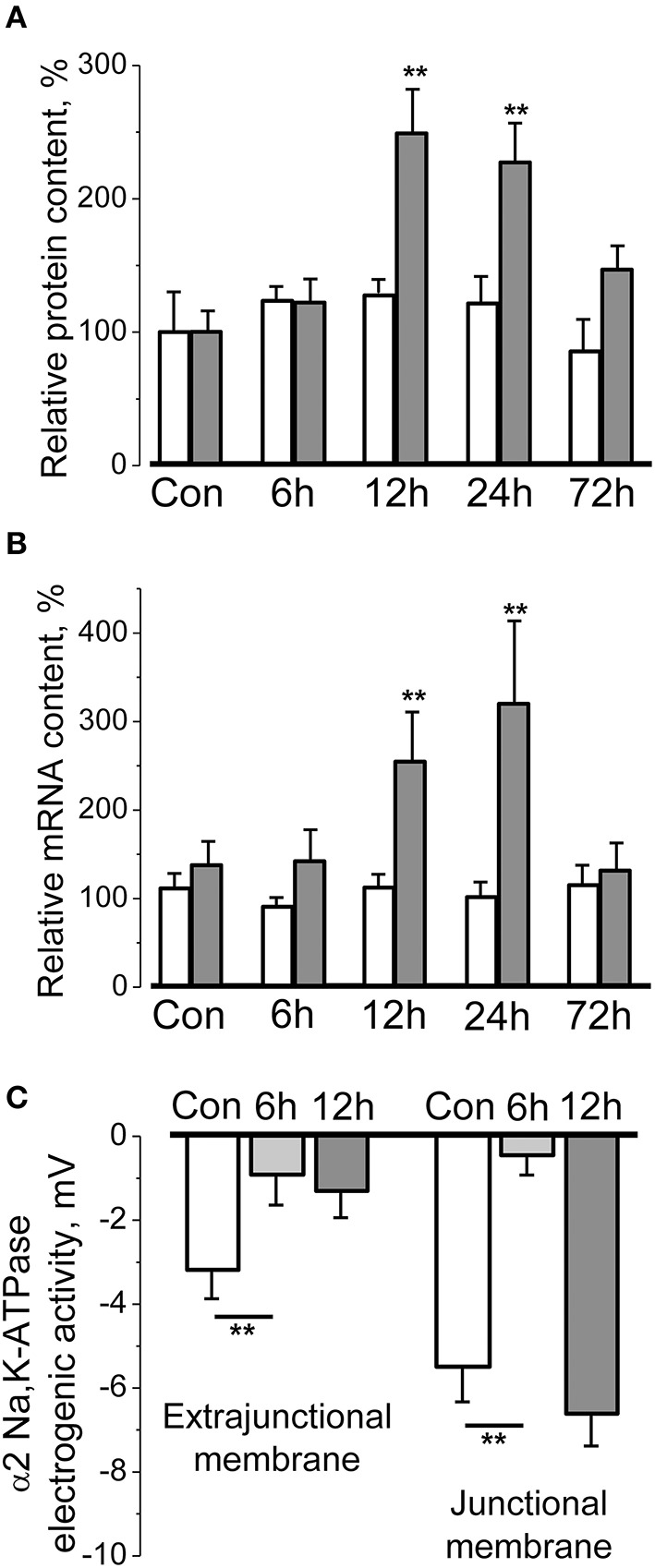
**Short-term hindlimb suspension specifically and transiently alters the α2 Na,K-ATPase protein content (A), mRNA content (B), and electrogenic activity (C) in rat soleus muscle**. **(A,B)** Relative α1 (white bars) and α2 (gray bars) Na,K-ATPase protein and mRNA contents in the homogenates from muscles of control rats and after 6–72 h of hindlimb suspension (data normalized to the average level of expression under control conditions). **(C)** The electrogenic transport activity of the α2 Na,K-ATPase isozyme measured in the extrajunctional and junctional membrane regions of muscle fibers. ^**^p < 0.01 compared to corresponding control. Modified from Kravtsova et al. (2015a, 2016).

The hindlimb suspension (HS) of rodents is a well-validated model for skeletal muscle disuse providing an insight into underlying mechanisms of disuse-induced atrophy (Thomason and Booth, 1990; Morey-Holton et al., 2005; Shenkman and Nemirovskaya, 2008; Giger et al., 2009; Baldwin et al., 2013). HS leads to progressive and marked atrophy of the postural skeletal muscles which becomes evident already after 3–7 days and is associated with dramatic remodeling (Shenkman and Nemirovskaya, 2008; Baldwin et al., 2013; Pierno et al., 2013; Ogneva et al., 2014) that include a decrease of resting membrane potential (Desaphy et al., 2001; Pierno et al., 2002; Krivoi et al., 2008; Tyapkina et al., 2009). This membrane depolarization was shown to be a result of decreased electrogenic activity of the α2 Na,K-ATPase isozyme (Krivoi et al., 2008). Moreover, it was recently shown that short-term muscle disuse (6–72 h of HS) transiently and isoform-specifically regulates the electrogenic activity, protein, and mRNA content of α2 Na,K-ATPase isozyme in rat soleus muscle (Kravtsova et al., 2015a, 2016) (Figures 1A,B). Importantly, electrogenic activity of the α2 Na,K-ATPase isozyme was altered by a decrease in enzyme activity rather than as a consequence of altered mRNA and protein contents or localization in the sarcolemma. The loss of α2 Na,K-ATPase electrogenic activity on extrajunctional membranes containing a majority of α2 pump cannot be compensated by increase of protein and mRNA contents observed after 12 h of HS. In contrast, a small subset of junctional α2 Na,K-ATPase demonstrated recovery (Figure 1C) suggesting that distinct pools of the α2 isozyme are differently regulated during HS. Importantly, acute low-intensity muscle workload restores function of both pools of the α2 Na,K-ATPase (Kravtsova et al., 2016).

These disuse-induced alterations in α2 Na,K-ATPase isozyme function and expression may involve PLM-dependent regulatory mechanism (Kravtsova et al., 2016). Muscle-specific auxiliary FXYD1 subunit, PLM is one of the most abundant phosphoproteins in skeletal muscles. PLM acts as a tissue-specific regulator of the Na,K-ATPase which suppresses enzymatic activity mostly by reducing Na^+^ affinity. Phosphorylation of PLM removes this inhibition and thereby increases the Na,K-ATPase pumping activity. Thus, protein kinases A (PKA) and C alter the PLM substrate affinity or turnover in cell- and Na,K-ATPase isoform-specific manner (Geering, 2008; Bossuyt et al., 2009; Pavlovic et al., 2013). In muscular tissues PLM associates with both α1 and α2 Na,K-ATPase isozymes (Crambert et al., 2002; Reis et al., 2005; Bossuyt et al., 2009; Heiny et al., 2010; Chibalin et al., 2012) where at least 30% of them are associated with the PLM (Rasmussen et al., 2008). Although the exercise-induced regulation of PLM was previously shown (Juel, 2009), the mechanism behind it remains to be elucidated. It was shown that acute HS increased PLM phosphorylation at Ser^63^ and Ser^68^ (Kravtsova et al., 2016). This is expected to stimulate the Na,K-ATPase and might be an earlier adaptive response directed to counteract the loss of enzyme activity. At the same time an increased abundance and association of PLM with the α2 Na,K-ATPase were shown to provide an opposite inhibitory effect and the net pump inhibition was achieved (Kravtsova et al., 2016).

A unique role of the α2 Na,K-ATPase isozyme in the adaptations to skeletal muscle disuse is supported by the studies in humans. Thus, chronic disuse resulting from spinal cord injury (Boon et al., 2012) or knee injury (Perry et al., 2015) significantly decreased the α2 Na,K-ATPase content in human skeletal muscles. These findings raise an interesting question whether the α2 Na,K-ATPase content or activity is regulated during other forms of disuse, e.g., sleep or treatment with muscle relaxants and anesthetics. It has been shown that electromyography (EMG) signal from soleus muscle disappears immediately after the onset of HS and remains dramatically low for several days (Ohira et al., 2002; De-Doncker et al., 2005). In contrast, different forms of periodic limb movement occurring during sleep (De Weerd et al., 2004) associate with brief EMG bursts and soleus muscle contractions (Eken, 1998). Accordingly, it was recently shown that a minimal low-intensity workload is able to restore electrogenic activity of the α2 Na,K-ATPase isozyme in soleus muscle of hindlimb-suspended rats (Kravtsova et al., 2016). These findings are in agreement with observation that limited physical activity is able to maintain abundance of the Na,K-ATPase in skeletal muscles of patients with partial spinal injury (Boon et al., 2012). Another potential experimental model to study the regulation of the Na,K-ATPase by muscle use could be hibernating animals, which overcome muscle atrophy despite prolonged disuse in dormancy.

Taken together, these results suggest that alterations specific for the α2 Na,K-ATPase precede disuse-induced skeletal muscle atrophy and indicate that different pools of this isozyme are regulated differently. Importantly, acute HS did not alter activity and content of the α1 Na,K-ATPase isozyme (Figures 1A,B). These findings are consistent with generally accepted hypothesis that ubiquitous α1 isozyme in skeletal muscle, as well as in other tissues, plays the main “house-keeping” role while the α2 Na,K-ATPase isozyme involved preferably in the regulation of cellular functions (Lingrel, 2010; Matchkov, 2010; Krivoi, 2012; Shattock et al., 2015). Specific regulation of the α2 Na,K-ATPase might be determined by its functional and molecular environment (Blaustein and Golovina, 2001; Lencesova et al., 2004; Song et al., 2006; Blaustein, 2013; DiFranco et al., 2015; Shattock et al., 2015) as well as by less stable than other Na,K-ATPase isozymes integration into the lipid membranes (Lifshitz et al., 2007; Kapri-Pardes et al., 2011).

## Interactions between Na,K-ATPase and nicotinic acetylcholine receptor

Inhibition of the Na,K-ATPase activity has profound effects on synaptic function associated with nerve endings membrane depolarization that stimulates release of neurotransmitters (Lichtstein and Rosen, 2001; Reich et al., 2004; Richards et al., 2007; Gulledge et al., 2013). However, it has recently become clear that the Na,K-ATPase functionally and molecularly interacts with a number of proteins and lipids to modulate synaptic, neuronal, and other cellular functions (Khatri and Man, 2013; Reinhard et al., 2013; Cornelius et al., 2015). Na,K-ATPase has been demonstrated to interact with dopamine (Hazelwood et al., 2008), AMPA (Zhang et al., 2009), δ-opioid (Deng et al., 2009), and adenosine A_2A_ (Matos et al., 2013) receptors. Moreover, functional interactions with glutamate transporters controlling glutamate uptake by astrocytes (Rose et al., 2009; Genda et al., 2011; Illarionava et al., 2014) and GlyT2 glycine transporter play an important role in glycinergic neurotransmission control (de Juan-Sanz et al., 2013).

Both the Na,K-ATPase and the nicotinic acetylcholine receptor (nAChR) are integral membrane proteins that play key roles in membrane excitation. A regulatory mechanism, where the nAChR and the Na,K-ATPase functionally interact to modulate the membrane potential, was shown in ganglion neurons (Park et al., 2010) and in skeletal muscles (Henning et al., 1994; Kragenbrink et al., 1996; Krivoi et al., 2003, 2006; Heiny et al., 2010). In ganglion neurons, micromolar concentrations of acetylcholine (ACh) induce fast depolarization through an activation of the nAChR followed by sustained hyperpolarization after ACh removal. This afterhyperpolarization is partly enabled by increase in Na^+^ entry which activates the Na,K-ATPase in concentration-dependent manner. It has been suggested that this afterhyperpolarization attenuates the firing rate of post-synaptic neurons acting as an auto-regulatory mechanism for neurons excitability (Park et al., 2010).

The nAChR oscillates between resting (micromolar affinity for agonist), open or desensitized (non-conducting state with nanomolar apparent affinity for agonist) conformations (Prince and Sine, 1999; Mourot et al., 2006). High concentrations of ACh promote channel opening following by spontaneous transitions into the desensitized state. Desensitization can also occur without channel opening and is favored by prolonged exposure to low concentrations of agonist. In skeletal muscle, specific binding of nicotinic agonists to the nAChR stimulates electrogenic transport by the Na,K-ATPase causing membrane hyperpolarization. An essential role of the α2 isozyme in this response has been identified (Krivoi et al., 2003, 2006; Heiny et al., 2010). In contrast to ganglion neurons, this effect was induced by nanomolar concentrations of nicotinic agonists (K_0.5_ ~ 30 nM for ACh) (Krivoi et al., 2006). Importantly, stimulation of the Na,K-ATPase activity did not require ion current through the open nAChR (Heiny et al., 2010). It can be induced by the nAChR desensitization alone in the absence of nicotinic agonist and reaches saturation when the nAChR is fully desensitized. Thus, the Na,K-ATPase activation may be triggered by non-competitive blockers of the nAChR, e.g., proadifen and QX-222, which promotes the desensitized states, and suppressed by tetracaine that stabilizes resting conformation of the nAChR (Heiny et al., 2010).

**Figure 2:**
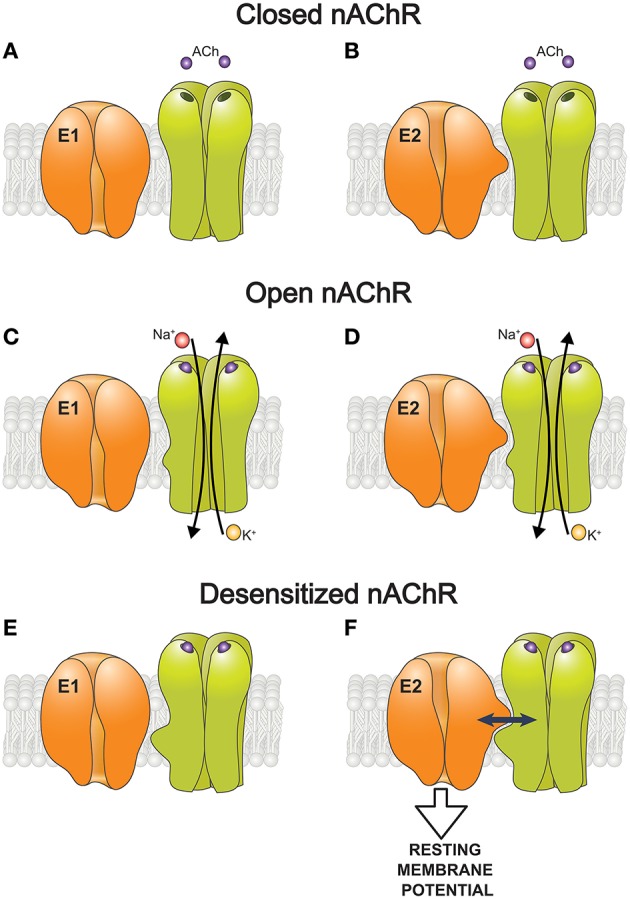
**A hypothetic scheme of the functional interaction between the nAChR and the α2 Na,K-ATPase isozyme that modulate the resting membrane potential. (A–F)** Schematic representation of how the nAChR in resting (closed), open, or desensitized conformations interacts with the Na,K-ATPase in E1 **(A,C,E)** and E2 **(B,D,F)** conformations. Only the desensitized state of the nAChR and E2 conformation of the Na,K-ATPase are capable interact functionally with each other **(F)**.

The nAChR/Na,K-ATPase interaction was demonstrated in a purified membrane preparation from *Torpedo californica*, enriched by the nAChRs and the Na,K-ATPase (Krivoi et al., 2006; Heiny et al., 2010). This preparation lacks transmembrane ionic gradients and many modulatory/associative proteins and factors presented in the intact cell. It was shown that binding of nanomolar concentrations of ouabain to the Na,K-ATPase modulates specific ligand interaction with the nAChR, and vice versa, suggesting a reciprocal modulation between these two proteins (Krivoi et al., 2006). Additionally, ouabain-induced conformational changes of the Na,K-ATPase enhance conformational transition of the nAChR into a desensitized state (Heiny et al., 2010). Accordingly, these findings suggest a mechanism by which the nAChR (in desensitized state) interacts with the Na,K-ATPase and stimulates its pumping activity. Taken into account that the binding of ouabain stabilizes the enzyme E2 conformation, it can be suggested that this conformation of the Na,K-ATPase and desensitized state of the nAChR are essential for the functional interaction between these proteins (Krivoi et al., 2006; Heiny et al., 2010; Krivoi, 2012; Figure 2).

Interaction between the nAChR and the Na,K-ATPase is expected to enhance muscle excitation in response to nanomolar concentrations (up to 50 nM) of non-hydrolyzed ACh which escaped hydrolysis by acetylcholinesterase, attributed to ACh released in non-quantal form (Nikolsky et al., 1994; Vyskocil et al., 2009) and remained in the synaptic cleft after nerve excitation. These nanomolar concentrations of ACh are insufficient to trigger any massive opening of the nAChR channels but it can selectively stimulate the α2 Na,K-ATPase isozyme leading to hyperpolarization of junctional membrane by ~2–4 mV (Heiny et al., 2010; Chibalin et al., 2012). Importantly, this local hyperpolarization keeps junctional membrane at a slightly more negative potential than extrajunctional regions of the same muscle fibers. This data suggests a mechanism by which the interaction between nAChR and α2 Na,K-ATPase isozyme maintains resting potential at voltage range where the Na^+^ channel inactivates slowly. This supports the excitability of junctional membrane during muscle use (Heiny et al., 2010).

This finding suggested that chronic *in vivo* exposure to nicotine, the concentration of which reaches hundreds of nanomoles during tobacco smoking (Benowitz et al., 1997) might produce long-term effects on the Na,K-ATPase and skeletal muscle electrogenesis. Experiments on rats chronically (for 21–31 days) exposed to nicotine delivered orally demonstrated that nicotine is able to modulate both α1 and α2 isozymes of the Na,K-ATPase in the diaphragm muscle. The regulatory effects include both stimulation of the α2 isozyme and inhibition of the α1 isozyme electrogenic activity leading to the net depolarizing effect. Increase in the α2 isozyme activity was accompanied with decrease in its content in the sarcolemma without change in total homogenate. The same nicotine treatment activated PKC and increased PLM phosphorylation suggested that cholinergic modulation of the Na,K-ATPase activity may utilize this regulatory pathway (Chibalin et al., 2012). Stable reciprocal interaction between the nAChR of neuronal type and the Na,K-ATPase was further confirmed in an insect nervous system (Bao et al., 2015). However, in contrast to skeletal muscle, the α2 Na,K-ATPase content decreased in homogenates of cerebral microvessels and brain tissues of rats chronically (for 14 days) exposed to nicotine using osmotic mini-pumps (Wang et al., 1994). The reasons of this contradiction are not clear; time- and use-dependence of chronic nicotine effects as well as high Ca^2+^ permeability and other features of neuronal nAChRs can be proposed.

It is established that the α2 Na,K-ATPase isozyme is enriched in end-plate membrane where it co-localizes with the nAChRs. It was also shown that the nAChRs and both α1 and α2 Na,K-ATPase isozymes co-immunoprecipitate with each other and with PLM and caveolin-3 (Heiny et al., 2010). Caveolin-3 is enriched at the neuromuscular junction (NMJ) where it co-localizes with the nAChR and promotes their clustering in the end-plate membrane. In congruence, the α subunit of nAChR has previously been shown to have a putative caveolin-binding motif (Hezel et al., 2010). Moreover, the caveolin/Na,K-ATPase interactions are also well-documented (Wang et al., 2004; Morrill et al., 2012). Since caveolin-3 is associated with caveolae in fully differentiated skeletal muscles (Galbiati et al., 2001) it suggests that the nAChR/α2 Na,K-ATPase interaction localizes in caveolae (Heiny et al., 2010). This spatially restricted complex is implemented by either direct protein-protein interaction or via additional adaptive molecular partners including lipids in the cholesterol-rich membrane microdomains, i.e., lipid rafts and caveolae. Direct molecular interactions between cholesterol and membrane receptors are shown. The role of cholesterol-rich lipid rafts as a signaling platform for the nAChRs clustering is well-established (Willmann et al., 2006; Zhu et al., 2006; Brannigan et al., 2010; Levitan et al., 2014). On the other hand, cholesterol plays an essential role in regulation of the Na,K-ATPase (Cornelius, 2008; Chen et al., 2009, 2011; Cornelius et al., 2015). It was recently shown that cholesterol chelating agent, methyl-β-cyclodextrin, eliminates local hyperpolarization of junctional membrane in rat diaphragm muscles through a selective decrease in the α2 Na,K-ATPase isozyme electrogenic activity (Kravtsova et al., 2015b). This data suggests the involvement of cholesterol in formation and function of the nAChR/α2 Na,K-ATPase complex.

Dystrophin is a cytoskeletal protein that localizes around entire sarcolemma membrane and provides scaffolding essential for stabilization of the nAChR clusters in the NMJ. Mice lacking dystrophin (i.e., X chromosome-linked mouse mutant, MDX) causes disruption of the NMJ and de-clustering of the nAChRs (Ghedini et al., 2008; Banks et al., 2009) as well as depolarization of plasma membrane due to loss of the Na,K-ATPase activity (Miles et al., 2011). The specific involvement of the α2 Na,K-ATPase isozyme in these changes has been suggested (Kravtsova et al., 2010). However, the participation of dystrophin and other potential molecular partners, such as spectrins and ankyrins (Williams et al., 2001; Lencesova et al., 2004; Mohler et al., 2005; Doi and Iwasaki, 2008) in the formation of the nAChR/α2 Na,K-ATPase complex remains to be elucidated.

## Cardiotonic steroids and cell survival

Several reports suggest that endogenous ouabain or ouabain-like compound changes the activity of α2 or α3 Na,K-ATPase isozymes that modulates glial and neuronal functions (Song et al., 2013) and may be involved in mood disorders (Lichtstein and Rosen, 2001; Goldstein et al., 2006, 2011). In addition, upon ouabain binding the Na,K-ATPase interacts with neighboring molecular environment to downstream a number of signaling intracellular pathways (Xie and Askari, 2002; Aperia, 2007; Li and Xie, 2009; Fontana et al., 2013; Reinhard et al., 2013). In different cell types, ouabain has dual effects to promote programmed cell death (Xiao et al., 2002; Kulikov et al., 2007; Blanco and Venugopal, 2016) and to protect against apoptosis (Isaev et al., 2000; Dvela et al., 2012). Nanomolar ouabain concentrations were also shown to stimulate viability and proliferation of NT2 cells, precursors for human neuronal cells, by a mechanism involving Erk1/2 activation (Dvela et al., 2012). Chronic intraperitoneal administration of low doses of ouabain significantly improves functional recovery following traumatic mouse brain injury (Dvela-Levitt et al., 2014).

Functional dysregulation of neuronal metabolism resulting from over-activation of glutamate receptors (GluRs) leads to neuronal death and underlies a variety of central nervous system disorders including stroke, neurodegenerative diseases, and spinal cord and brain injuries. The excitotoxic stress response starts with a free intracellular Ca^2+^ overload which is the most important of apoptosis (Khodorov, 2004). A similar mechanism of neuronal dysfunction and cell apoptosis is induced by micromolar ouabain (Kulikov et al., 2007; Bolshakov et al., 2012). However, some animal experimental studies have shown that low-doses of CTS provide neuroprotection against ischemia (Wang et al., 2006; Oselkin et al., 2010). This anti-apoptotic action of low ouabain was described when kainic acid and ouabain were injected into the rat brain *in vivo* (Golden and Martin, 2006).

Recently it was shown that ouabain at subnanomolar concentrations can prevent GluR agonist-induced apoptosis in primary culture of rat cortical neurons (Bolshakov et al., 2012; Sibarov et al., 2012). Apoptotic injury was prevented when the agonists were applied together with 0.1–1 nM ouabain resulting in survival of neurons in this model of excitotoxicity. Accordingly, ouabain modulated the level of anti-apoptotic protein Bcl-2, an important regulator of mitochondrial function and energy metabolism, involved in many vital cell processes (Zheng et al., 2015). Similar anti-apoptotic effects of low ouabain doses have been shown to be associated with enhanced production of Bcl-2 in *in vivo* rat model for neurodegeneration (Golden and Martin, 2006). In cultured rat cortical neurons ouabain also prevented the increase in frequency of spontaneous excitatory postsynaptic current and the intracellular Ca^2+^ overload induced by 240-min exposure to 30 μM *N*-methyl-D-aspartate (NMDA). These effects were absent in the presence of KB-R7943, the plasma membrane NCX inhibitor (Sibarov et al., 2012). In addition, the postsynaptic epileptiform currents, reflecting periodical asynchronous glutamate release associated with elevations in intracellular Ca^2+^ concentration, were found to be suppressed by 1 nM ouabain (Sibarov et al., 2014). Ouabain was, however, found to have a bimodal effect; including anti-apoptotic action in excitotoxic stress in the concentration range from 0.1 to 1 nM, and toxic action at concentrations 10 nM–30 μM (Bolshakov et al., 2012).

**Figure 3:**
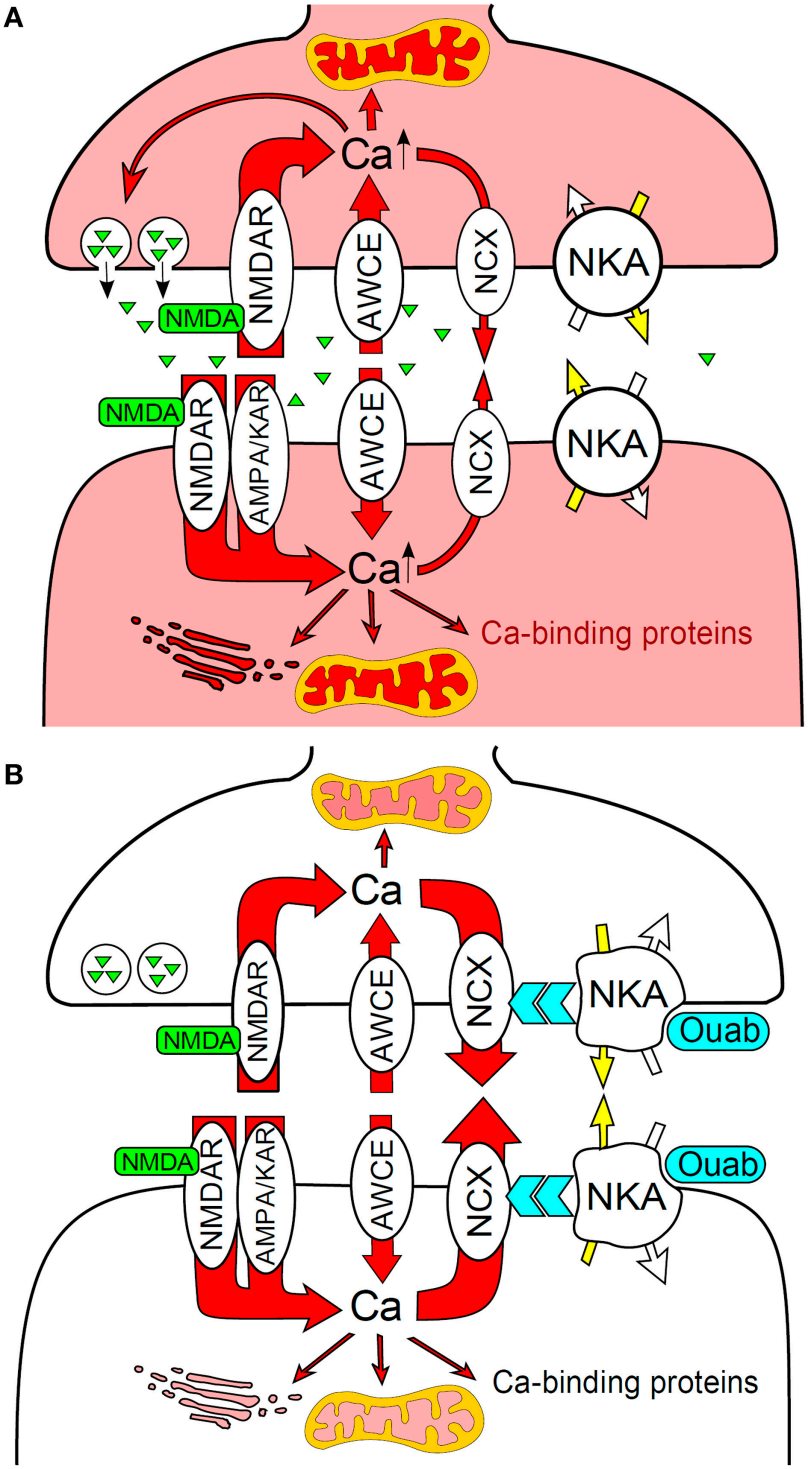
**Na,K-ATPase functionally interacts with the plasma membrane NCX to prevent Ca^2+^ overload and neuronal apoptosis during excitotoxic stress. (A)** NMDA activates presynaptic and postsynaptic NMDA receptors resulted in intense Ca^2+^ entry and intracellular accumulation. **(B)** Ouabain binding to Na,K-ATPase modulates the NCX that accelerates Ca^2+^ extrusion and prevents neurons from Ca^2+^ overload. AMPA/KAR, AMPA and kainic acid receptors; AWCE, alternative to GluRs ways of Ca^2+^entry; NKA, Na,K-ATPase. Modified from Sibarov et al. (2012).

It was suggested (Sibarov et al., 2012) that during excitotoxic insults ouabain accelerates Ca^2+^ extrusion from neurons via functional interaction between the Na,K-ATPase and the NCX (Figure 3). Since ouabain inhibits neuronal α3 Na,K-ATPase isozyme in rats at concentrations that exceed those having anti-apoptotic effects (i.e., 0.1–1 nM) (Richards et al., 2007) it can be suggested that this neuroprotective effect takes place via signaling pathways and does not directly depend on ion translocation by the Na,K-ATPase (Sibarov et al., 2012). Accordingly, circulating endogenous ouabain concentration in rat blood plasma and cerebrospinal fluid varies between 0.1 and 0.74 nM (Dobretsov and Stimers, 2005). This signaling hypothesis has been further supported by a demonstration in the crystal structure of Na,K-ATPase in high-affinity binding state for ouabain with equilibrium dissociation constant of ~1 nM (Ogawa et al., 2009). Taken together this data suggests the novel function of the Na,K-ATPase as a neuroprotective molecule that triggers signaling pathways upon binding of endogenous ouabain or ouabain-like compounds by highly conserved binding site.

Finally, neuroprotective effects of exogenous CTS were shown *in vivo* (Golden and Martin, 2006; Wang et al., 2006; Oselkin et al., 2010). However, if endogenous CTS are already neuroprotective, then exogenously administered ouabain should have no additional effect. This opens interesting and provocative question whether the neuroprotective effects of endogenous CTS are not saturated at physiological conditions due to different properties compared to exogenous analogs. An alternative explanation suggests that different regulatory pathways are triggered and the neuroprotective effects of endogenous and exogenous CTS are not additive.

## Concluding remarks

The importance of Na,K-ATPase in various cell functions recently received new attention. It became clear that the functional role of the Na,K-ATPase can only be considered in a complex environment at the subcellular, cellular and multicellular levels where the Na,K-ATPase is structurally and functionally linked to other membrane transporters, cytoskeleton proteins and signaling molecules. We are currently only at the beginning of our understanding of these complexities. Future studies of these signalosomes, organized around specific isozymes of the Na,K-ATPase, will lead to a conceptually new view on cell physiology and will provide novel targets in treatment of several life-threatening diseases, e.g., psychiatric diseases, hypertension, and heart failure.

## Author contributions

Conception and design, analysis and interpretation of data, drafting the article, article revision and approval of the final version of the manuscript: VM and IK contributed equally.

### Conflict of interest statement

The authors declare that the research was conducted in the absence of any commercial or financial relationships that could be construed as a potential conflict of interest.
